# Ecological Implications of Germination Temperature on Native and Invasive *Rumex* Spp.

**DOI:** 10.1002/pei3.70045

**Published:** 2025-03-27

**Authors:** Michaela Jungová, Martina Kadlecová, Vilém Pavlů, Leona Leišová‐Svobodová, Pavel Svoboda, Zdenka Martinková

**Affiliations:** ^1^ Czech Agrifood Research Center Prague 6‐Ruzyně Czech Republic; ^2^ Department of Applied Ecology, Faculty of Environmental Sciences Czech University of Life Sciences Prague Prague 6‐Suchdol Czech Republic; ^3^ Department of Biology and Ecology Technical University of Liberec Liberec Czech Republic

**Keywords:** alpine dock, broad‐leaved dock, invasive plant, long‐leaved dock, mountain plants, weeds

## Abstract

*Rumex alpinus* L. ( *R. alpinus* ) is a non‐native invasive plant in Czech mountain regions, altering ecosystem structure and function in protected areas. *Rumex obtusifolius* L. ( *R. obtusifolius* ) is a native species and a problematic weed in Czech meadows, while *Rumex longifolius* DC. ( *R. longifolius* ) is characteristic of Fennoscandia and widespread in northern and central Europe. This study explores temperature‐driven germination patterns in *R. alpinus* , *R. obtusifolius* , and *R. longifolius* and also focuses on potential differences across populations of *R. alpinus* . The hypothesis suggests that *R. alpinus* is not established in lowland areas due to temperature limitations during germination. A second experiment evaluates the influence of native and non‐native localities on *R. alpinus* seed germination. The primary experiment was conducted at 6°C, 12°C, 18°C, 24°C, 29°C, and 35°C in a climate chamber, while the second experiment was performed at 24°C for 14 days. Contrary to expectations, *R. alpinus* exhibited the highest germination rate across all temperatures. In the second experiment, germination rates varied significantly, with a positive correlation between germination success and transition from Alpine to Czech localities. The highest and fastest germination was observed in seeds from the Krkonoše Mountains, where *R. alpinus* is an invasive plant species.

## 1 Introduction

*Rumex alpinus* commonly referred to as ‘alpine dock,’ is native to the high mountains of Europe, the Balkan Peninsula, the Caucasus, and the Apennine Peninsula, including Armenia and northern Anatolia (Raycheva and Dimitrova 2007; Delimat and Kiełtyk 2019). It has been identified as a long‐term problematic weed, spreading due to its ability to outcompete native species and leading to ecosystem imbalances (Stachurska‐Swakoń 2009; Šťastná et al. 2010; Delimat and Kiełtyk 2019). Its expansion is particularly noticeable in high alpine herb communities, such as *Rumicetum alpini* Beger (1922), and in deforested areas of the subalpine zone (Bohner 2005). *R. alpinus* thrives in alpine environments due to the favorable climatic and light conditions that prevail above the timberline (Nagelmüller et al. 2017).

The distribution of *R. alpinus* in European mountain areas has expanded with the advent of agricultural settlement and anthropogenic colonization, reaching lower mountain ranges (e.g., the Krkonoše [Giant] Mountains in the Czech Republic) where it is now considered non‐native. It has been described as a non‐native, neophyte plant (Kopecký 1973; Chytrý et al. 2021) and an invasive species (Šťastná et al. 2010; Pyšek et al. 2012; Jungová, Asare, et al. 2023). Within these novel habitats, *R. alpinus* has been shown to thrive under optimal conditions, including abundant nutrients and sunlight, often facilitated by free‐range grazing (Červenková and Münzbergová 2009; Náglová 2014). One of the key factors underlying its competitive success is its prolific production of seeds with high tolerance and fertility, ranging from 1500 to 5000 per flowering plant (Šťastná et al. 2010). Furthermore, *R. alpinus* maintains a substantial seed bank, with seeds remaining dormant for extended periods, sometimes up to 80 years (Darlington and Steinbauer 1961; Klimeš 1992; Martinková et al. 1999).

Despite its adaptability, *R. alpinus* is only found at altitudes below 600 m in the Czech Republic and 1300 m in the Alps (Klimeš 1992; Bohner 2005; Šťastná et al. 2010; Wild et al. 2019; Jungová, Jurasová Müllerová, et al. 2023). The reasons for this distribution pattern are not yet fully understood. Given the species' dependence on nutrient‐rich habitats (Jungová, Asare, et al. 2023) and its capacity for expansion via water dispersal (Červenková and Münzbergová 2009), it is possible that it may colonize lower elevations in the future. Consequently, from an ecological and conservation perspective, gathering comprehensive data on this species is crucial, with a particular emphasis on its seed germination under elevated temperatures. This is of particular relevance in the context of ongoing climate change and the associated warming that is occurring, which has the potential to influence the species' distribution. Paradoxically, while warming may facilitate its spread to lower elevations, it could also contribute to its decline in mountainous regions due to heat stress.

In the context of climate change, it is imperative to understand the impact of temperature on plant species distribution (Kadlecová et al. 2024). The study investigates how temperature affects the germination of *R. alpinus* , providing insights into its current range and future spread under changing climatic conditions. To this end, the study aims to examine the germination characteristics of *R. alpinus* across different temperature conditions to assess whether temperature limits its establishment in lowland habitats.

To investigate the role of temperature in the distribution of *R. alpinus* , we conducted an experiment to explore germination differences among its seeds and their potential implications for species distribution. To provide a comparative framework, we included *R. obtusifolius* (broad‐leaved dock), a closely related species with a similar geographic range and anatomical characteristics. *R. obtusifolius* is predominantly found in lowland areas, where it represents a widespread issue in grassland ecosystems (O'Donovan et al. 2023). However, it also occurs at higher elevations, making it a suitable reference species for examining the potential germination constraints of *R. alpinus* . The aim was not to assess direct competition between these species but rather to highlight differences in germination strategies that could influence their respective distribution patterns (Kubínová and Krahulec 1999). Additionally, we examined *R. longifolius* (long‐leaved dock), which is native to northwestern to southeastern Fennoscandia and widespread in the northern British Isles and the Pyrenees (Rechinger 1990). *Rumex longifolius* is closely related to *R. obtusifolius* and is non‐invasive in the Czech Republic, yet its expansion at higher elevations suggests potential adaptation to cooler conditions (Rechinger 1990).

Building on previous findings, we conducted an additional experiment to examine differences in seed germination between *R. alpinus* populations from their native range (the Alps) and non‐native populations in the Czech Republic (Jungová, Jurasová Müllerová, et al. 2023). This study takes an exploratory approach to assess how germination patterns vary across different environments, aiming to identify potential limiting factors beyond temperature alone. As the first detailed investigation into the germination behavior of *R. alpinus* , it provides novel insights into the ecological conditions influencing its establishment and potential spread. Understanding these germination patterns is crucial for developing effective strategies to manage and mitigate the invasive potential of this species.

The objectives of this study are as follows:

1. To determine the impact of temperature on the success and timing of germination in *R. alpinus* , *R. obtusifolius* , and *R. longifolius* .
2. To compare interspecific differences in germination responses among these species.
3. To evaluate whether high temperatures impose a physiological constraint that limits *R. alpinus* expansion into lowland environments.
4. To investigate differences in germination responses between *R. alpinus* seeds from native and non‐native locations.

By addressing these questions, we seek to provide insights into the potential effects of climate change on species distribution and invasive dynamics.

## 2 Material and Methods

### 2.1 The Study Species

This study focuses on the germination traits of three closely related *Rumex* species, which differ in their ecological requirements and distribution patterns.

*Rumex alpinus* is primarily found in full light, but it can also survive in light shade (Ellenberg et al. 1992; Klimeš 1992; Šťastná et al. 2010). However, it is unable to persist in newly established forests despite the presence of sufficient water (Červenková and Münzbergová 2009). The germination rate of *R. alpinus* remains unaffected when far‐red radiation is filtered out, suggesting that in a dense leaf canopy, light intensity, rather than light quality, serves as the limiting factor (Bucharová 2003). Only a small percentage of seeds (3%–5%) germinate in complete darkness at room temperature (Klimeš unpublished data; Šťastná et al. 2010).

*Rumex obtusifolius* is native to central Europe and has emerged as a problematic weed due to its remarkable adaptability to mowing management in production grasslands (Zaller 2004; Vondráčková et al. 2014; Suter et al. 2023), and its prolific seed production contributes to the long‐term persistence of its seeds in the soil seed bank (Hrdličková et al. 2011; Hejcman et al. 2012). Holm et al. (1977) reported approximately 60,000 seeds per plant for *R. obtusifolius* .

*Rumex longifolius* is found in a variety of semi‐natural habitats, is adapted to high altitudes, and spreads by seed. As a non‐native species in the Czech Republic (Pyšek et al. 2012; Chytrý et al. 2021), the distribution of *R. longifolius* is mainly limited to mountainous areas (Kubínová and Krahulec 1999) and typically grows near human settlements, pastures, and rivers (Holm and Korpelainen 1999; Kubínová and Krahulec 1999). *Rumex longifolius* is adapted to a short vegetative season (90 to 120 days) with an average temperature exceeding 10°C (Walter and Lieth 1967). The species can produce ripe achenes in less than 3 to 4 months, exhibiting a high germination rate (Kubátová 1994); this enables it to invade new habitats rapidly and increase the number of individuals at the sites.

### 2.2 Collection of Plant Material

**FIGURE 1:**
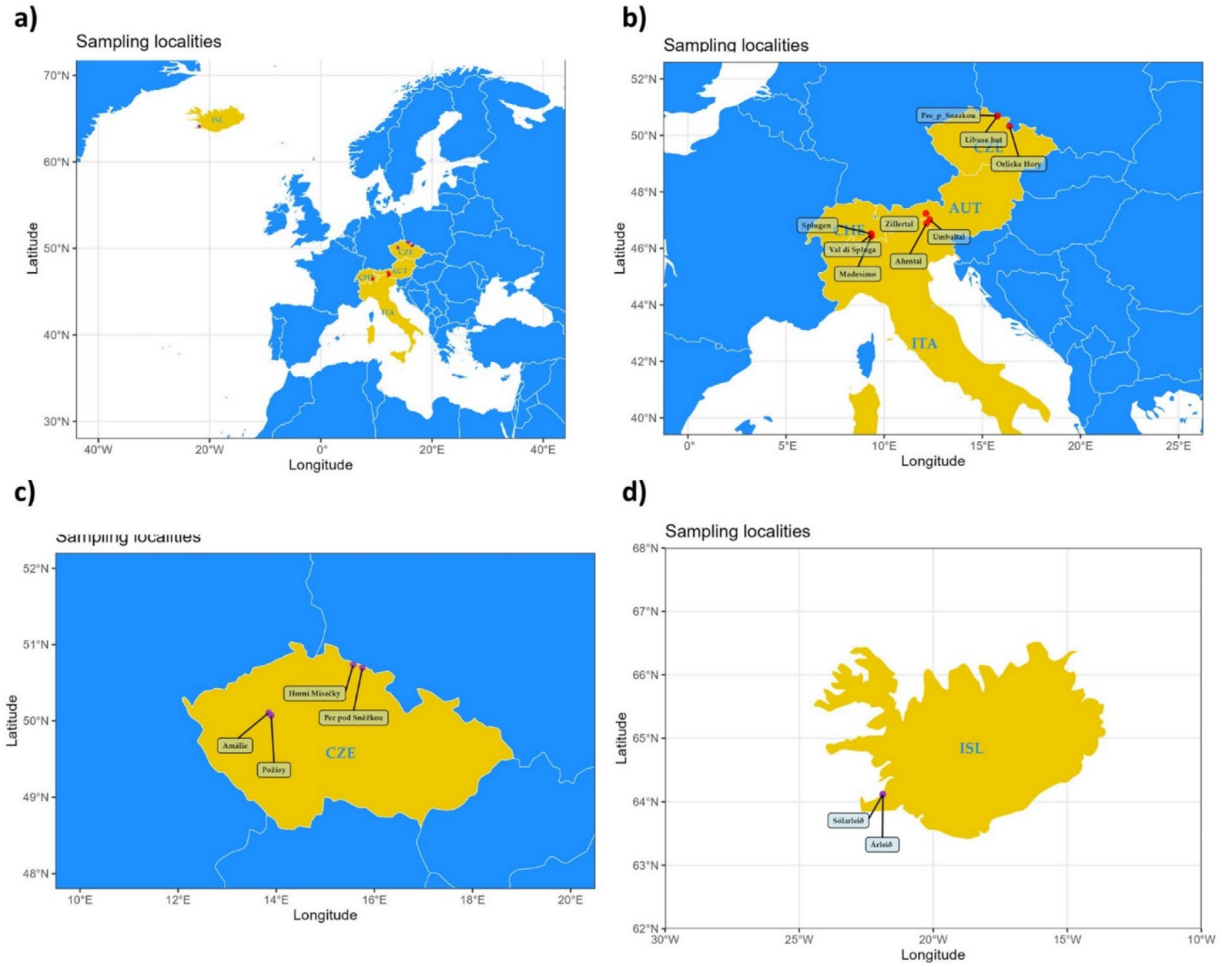
Location of studied localities in the a) Czech Republic (CZE) Austria (AUT); Italy (ITA); Switzerland (CHE); and Iceland b) Krkonoše Mountains‐ Libuše hut, Pec pod Sněžkou, and Eagle Mountains (Orlické hory); Austria —Umbaltal, Zillertal; Italy —Ahrntal, Madesimo, Val di Spluga; Switzerland ‐ Splügen; c) Czech Republic ‐ Horní Mísečky, Pec pod Sněžkou, Amálie, and Požáry, d) Iceland (ISL)—Árleið, Sólarleið.

The seeds of three *Rumex* spp. ( *R. alpinus* , *R. obtusifolius* , and *R. longifolius* ) were collected from various European locations (Figure 1a) and subjected to the experiment in a controlled laboratory setting. The seeds of *R. alpinus* were collected from localities in the Krkonoše Mountains; the first locality was the eutrophic grassland of Horní Mísečky (50°44′2″ N, 15°34′5″ E), and the second was the ruderal locality below the hotel Horizont in Pec pod Sněžkou (50°41′46″ N, 15°44′8″ E) (Figure 1c). The seeds of *R. obtusifolius* were collected in Lány, a village in the Central Bohemian Region about 20 km west of Prague, in the animal production center called Amálie (50°06′22.0″ N 13°50′43.9″ E) and the center called Požáry (50°04′14.6″ N 13°53′50.3″ E) (Figure 1c). The seeds of *R. longifolius* were collected in two grassland localities in the city of Reykjavik (Iceland): Sólarleið (64°7′4.466″ N 21°52′43.380″ W) and Árleið (64°6′58.3″ N 21°49′24.1″ W) (Figure 1d). As *R. longifolius* is not native to the Czech Republic (Pyšek et al. 2012) and only occurs in a few locations in the Giant Mountains (Kubínová and Krahulec 1999), seeds of this species were not found in the specific year of 2018 in the area under study, which was likely due to the protective management in the protected area. As a result, *R. longifolius* seeds were collected during an expedition to Iceland and subsequently used in this study.

### 2.3 Germination Experiment With *R. alpinus* , *R. obtusifolius* , and *R. longifolius* Seeds

The seeds of *Rumex* spp. were collected between September and October 2018. Seeds were sampled before dispersal from dry shoots, separately from five randomly selected plants at each locality deemed the most viable and fully mature. The experiments utilized freshly harvested seeds, necessitated the air‐drying of the seeds at room temperature (approximately 20°C) for 1 week. Subsequently, the seeds underwent a dark stratification period at 5°C for 6 weeks, which mimicked the winter conditions typically observed in central Europe (Mandák et al. 2006; Kołodziejek 2019). The perianth was removed manually by rubbing. The germination experiment was conducted with five replicates for each temperature and each *Rumex* spp., comprising 50 randomly selected but the same size seeds in five Petri dishes. The 50 seeds from each replicate were placed into a Petri dish of 10 cm diameter on filter paper moistened with 5 mL of tap water and placed in a climate chamber for the germination experiments. Six temperatures, as proposed by Shen et al. (2008), but with a minor revision, were set at the same time in each climate chamber (BINDER, Tuttlingen, Germany), with a specific temperature being applied.

The seeds were watered in the climate chamber with tap water on a regular basis every second day. The humidity level was maintained at 65%. The seeds germinated in a photoperiod of 4 h of light and 20 h of darkness, a period that proved conducive to the germination of non‐dormant seeds according to Martinková and Honěk (2000); Honěk and Martinková (2002). Seeds were recorded as germinating when the protruding radicle was ≥ 1 mm in length. The number of germinating seeds was recorded and removed at 24‐h intervals for a period of 29 days (Martinková and Honěk 2000). Seeds that did not germinate during the course of the experiment were returned to the 25°C climate box to determine if they were dead. However, 80% of these seeds germinated and were used in another experiment (Jungová, data not published).

### 2.4 Locality Germinated Experiment of *R. alpinus*

This experiment was conducted as a follow‐up to the previous one to identify potential differences between the plants of the *R. alpinus* species and the location. The parameters employed in this experiment differed from those used in the previous experiment, as we had gained more experience with the temperature and light regime that suits *R. alpinus* (based on the above experiment and our other unpublished study). As a non‐native invasive species in the Czech Republic (Pyšek et al. 2012; Wild et al. 2019; Chytrý et al. 2021), the investigation aimed to explore the germination patterns of seeds from native (Alpine) and non‐native (Czech Mountains) localities (Figure 1b). The study encompassed locations across four geographical regions in Western and Central Europe: the Krkonoše and Eagle Mountains in the Czech Republic and the Tyrolean and Lombardy Alps in Austria and Italy, respectively (Figure 1b). In the Alps, *R. alpinus* was collected in the approximately same vegetation zone as in the Czech mountains, despite the difference in altitude (Chytrý 2012; Divíšek et al. 2014). However, the climatic conditions and the locality of species with similar compositions should be approximately similar (Stachurska‐Swakoń 2009; Delimat and Kiełtyk 2019). The seeds of *R. alpinus* were collected between August and September 2019 in the Czech and Alpine Mountains (Figure 1b, Table 1). Seeds were sampled from dry shoots before dispersal, separately from five selected plants at each locality (a distance of 100 m was maintained between plants to prevent the collection of a single genotype) that were deemed to be the most viable and fully mature. Our experiments utilized freshly harvested seeds. The seeds were first air‐dried at room temperature (approximately 20°C) for 1 week. They were then subjected to a 6‐week dark stratification period at 5°C to simulate the winter conditions typical of Central Europe (Mandák et al. 2006; Kołodziejek 2019). The perianth was manually removed by rubbing. The germination localities experiment was conducted with four replicates for each temperature and for each *Rumex* spp. The seeds were randomly selected from a population of 50 seeds of similar size and were placed in five Petri dishes. The seeds of each replicate were placed into a Petri dish of 10 cm diameter on filter paper moistened with 5 mL of tap water and placed in a climate chamber BINDER (Tuttlingen/Germany), where germination experiments were conducted. The temperature regime was 24°C during the ‘day’ (16 h of light) and 15°C during the ‘night’ (8 h of darkness) corresponding to summer conditions, with a relative humidity of 65%. The seeds were watered with tap water at regular intervals every second day. The number of germinated seeds was counted and removed at 1‐day intervals for 14 days, provided the protruding radicle was at ≥ 1 mm in length. Seeds that did not germinate but exhibited the presence of a white embryo after being pinched with forceps were deemed viable (Baskin and Baskin 1985).

**TABLE 1:** Description of the localities where the germinated experiment of *R. alpinus* was conducted is provided, including the Krkonoše Mountains (Czech Republic) and the Alps (Austria, Italy).

Name of locality	Latitude	Longitude	Region	Type of locality	Altitude (m a.s.l.)
Libuše hut	50°41′19″ N	15°46′43″ E	Krkonoše_Czech Republic	Stream banks	750
Pec pod Sněžkou	50°41′46″ N	15°44′80″ E	Krkonoše_Czech Republic	Chalets	815
Eagle Mountains	50°19′34″ N	16°23′10″ E	Eagle Mountains_Czech Republic	Chalets, ski slope	1015
Ahrntal	46°53′1″ N	12°9′42″ E	Alps_Tyrol_Italy	Pasture	1450
Umbaltal	47°0′59″ N	12°19′15″ E	Alps_Tyrol_Austria	Pasture	1500
Zillertal	47°14′21″ N	12°7′39″ E	Alps_Tyrol_Austria	Ski slope, chalets	1650
Splügen	46°31′13″ N	9°19′50″ E	Alps_Lombardy_Switzerland	Next the road	1500
Madesimo	46°26′13″ N	9°21′27″ E	Alps_Lombardy_Italy	Ski slope	1600
Val di Spluga	46°28′70″ N	9°20′55″ E	Alps_Lombardy_Italy	Next the road	1700

### 2.5 Data Analysis

Germination dynamics were modeled using a three‐parameter logistic function:

$$G\left(t\right)=\frac{a}{1+\exp\left(-k\cdot \left(t-t_{50}\right)\right)}$$

where *G* (*t*) represents cumulative germination (%) at time *t* (days), *a* is the maximum germination (asymptote), *k* describes the slope of the curve (germination rate), and *t*_50_ denotes the time to reach 50% of maximum germination.

For each species (or locality), daily cumulative germination values were averaged across replicates to obtain mean germination percentages for each day. The logistic function was then fitted to these mean germination curves using non‐linear least squares regression. Curve fitting was performed in R (R Core Team 2024) using the *nlsLM* function from the *minpack.lm* package (Elzhov et al. 2023). Initial parameter estimates were derived directly from the observed data: *a* was set to the maximum observed germination, and *t*_50_ was initialized as the first day when germination reached or exceeded 50% of the final germination. The rate parameter *k* was initialized at 0.1.

Repeated measures ANOVA was applied to assess the germination dynamics of *Rumex* spp. (in five replications) across different temperature regimes (6°C, 12°C, 18°C, 24°C, 29°C, and 35°C). The number of germinated seeds at a given time was used as the response variable, while temperature regimes were treated as predictors. Figure 3, which visually summarizes germination trends within each species, was similarly evaluated statistically (Table 2).

**TABLE 2:** Repeated measures ANOVA results: Time × species for each temperature treatment.

Treatment	df	*F‐*statistic	*p*
6°C	58, 348	11.12	0.001
12°C	58, 348	60.41	0.001
18°C	58, 348	45.00	0.001
24°C	58, 348	15.61	0.001
29°C	58, 348	9.50	0.001
35°C	28, 168	17.28	0.001

Abbreviation: df = degrees of freedom.

**FIGURE 2:**
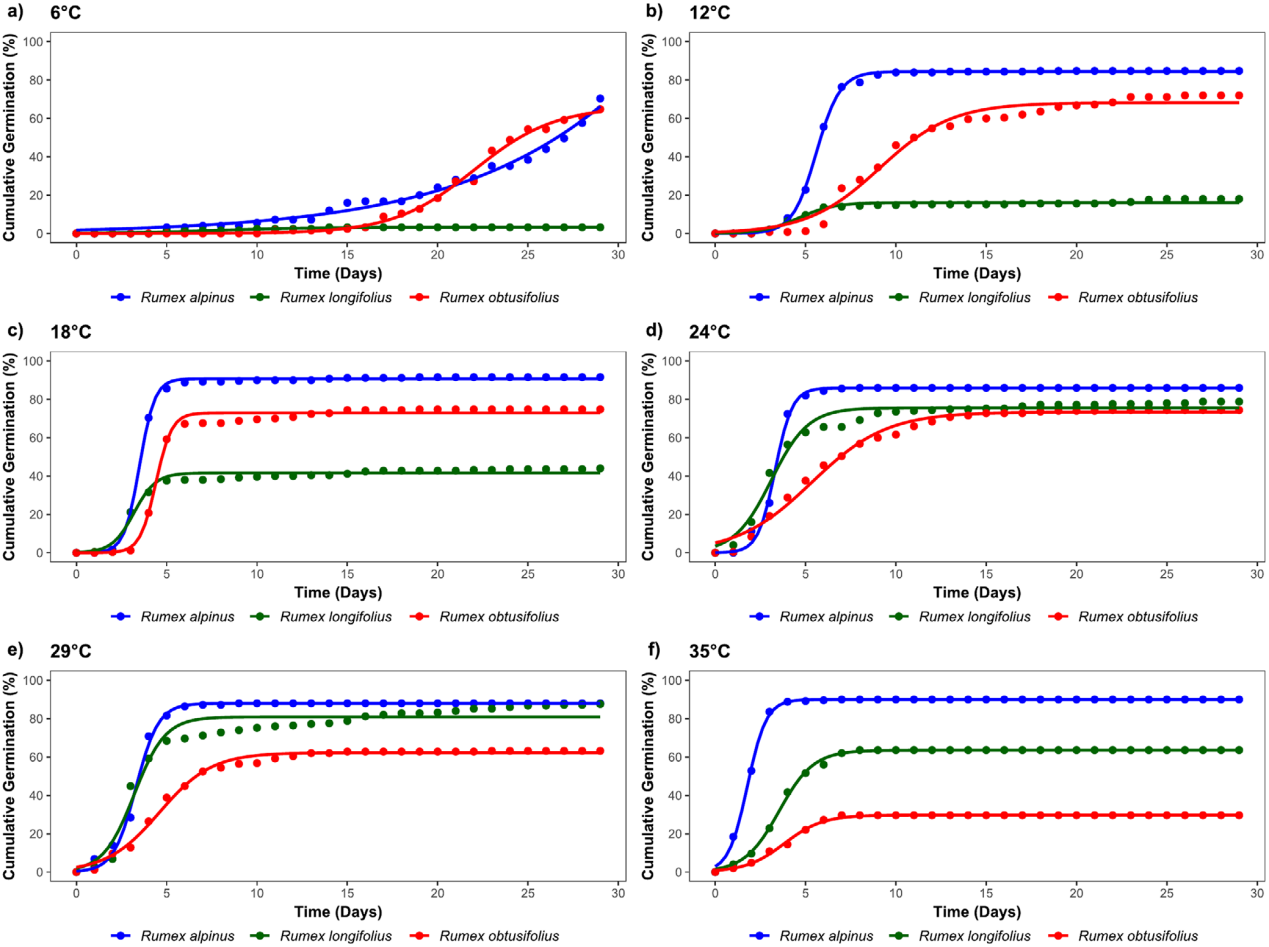
Cumulative germination (%) of three *Rumex* species over 29 days at different temperatures. Each subplot (a–f) represents a different temperature condition (6°C–35°C). Points indicate mean cumulative germination values from all replicates, while solid lines represent fitted sigmoidal growth curves, modeled using a three‐parameter logistic function. Different colors denote different species: *Rumex alpinus* (blue), *Rumex obtusifolius* (red), and *Rumex longifolius* (green).

Different temperature regimes were included as predictors. Table S1 of GR for all *Rumex* spp. at different temperatures is provided for the days with faster germination, specifically days 1 to 10, spanning temperatures from 12°C to 35°C. As germination occurred at a later stage, at 6°C, the temperature is not included in the table. The purpose of this table is to provide further clarification of the GR graphs (Figure 2), given that the graphs may not be entirely clear. The table is limited to day 10, as although the GR in the temperatures 12°C and 18°C was observed to increase at *R. obtusifolius* and *R. longifolius* until the 20th day, the effect was not as pronounced.

To compare final germination percentages across localities, mean germination values on day 14 of the experiment were calculated. Data were analyzed using a one‐way analysis of variance (ANOVA) with locality as the factor, followed by Tukey's HSD test to determine statistically significant differences between localities (*p* ≤ 0.05). Additionally, the average number of days required to reach maximum germination was calculated for each locality. The results are presented as a bar graph with error bars representing the standard error of the mean (SEM), and a gold line indicating the mean number of days to reach maximum germination.

All analyses were performed using R software version 4.4.0 (R Core Team 2024) and Statistica 13.3 (www.statsoft.com), with differences considered significant at *p* ≤ 0.05.

## 3 Results

### 3.1 Germination Experiment With *R. alpinus* , *R. obtusifolius* , and *R. longifolius* Seeds

To analyze germination patterns across different temperatures, a three‐parameter logistic function was fitted to cumulative germination data, providing a sigmoidal model for comparing germination responses (Figure 2). This model allows for a more precise interpretation of GR, capturing key parameters such as the time to reach 50% germination (*t*_50_) and maximum germination capacity (FG).

**FIGURE 3:**
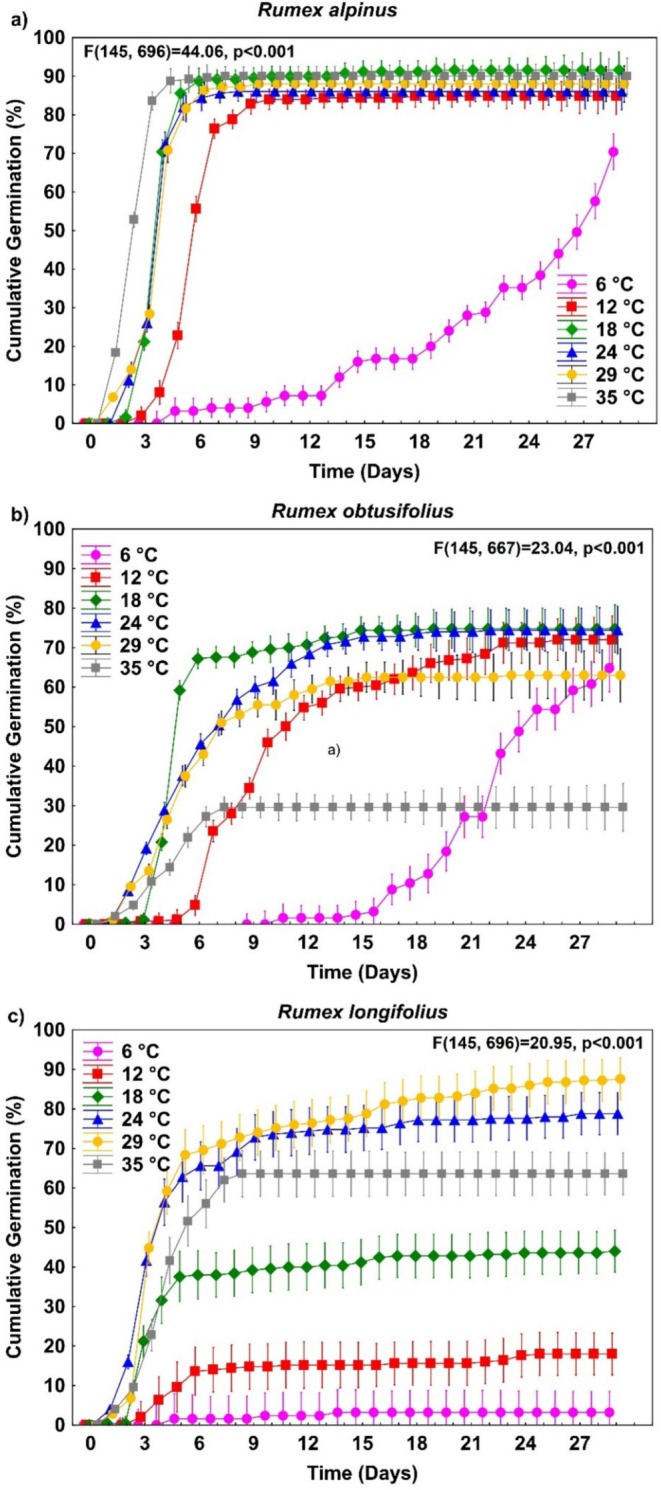
Time course of temperature regimes and germination rate in three *Rumex* species a) Rumex alpinus, b) Rumex obtusifolius, and c) Rumex longifolius, showing significant differences between species across all temperatures (repeated measures ANOVA, *p* < 0.001). Vertical bars represent ± standard errors.

At 6°C, germination was observed primarily in *R. alpinus* and *R. obtusifolius* , with both species reaching similar FG values of approximately 70% (Figure 2a). Germination onset was earlier in *R. alpinus* ; however, its progression was slower compared to *R. obtusifolius* . In contrast, *R. longifolius* exhibited minimal germination at this temperature, with an FG below 5% (Figure 2a, Figure 3c).

At 12°C, *R. alpinus* and *R. longifolius* began germination at a similar time; however, *R. alpinus* exhibited a significantly faster germination rate (Figure 2b). The *t*_50_ for *R. obtusifolius* was delayed compared to *R. alpinus* , but FG reached 75% (Figures 2b and 3b). *R. longifolius* displayed the slowest germination rate, with FG reaching only 18% (Figure 2c).

At 18°C, germination was most rapid, with *R. alpinus* and *R. longifolius* exhibiting the highest GR on day 2. However, *R. longifolius* had the lowest FG (44%) among all species (Figure 2c). The FG of *R. alpinus* reached 92%, with a large proportion (60%) of seeds germinating by day 3 (Table S1).

At 24°C, *R. alpinus* and *R. longifolius* exhibited similar germination onset, whereas *R. obtusifolius* showed a slower GR (Table S1). The highest FG was recorded for *R. alpinus* (86%), followed by *R. obtusifolius* (79%) and *R. longifolius* (74%) (Figure 2d).

At 29°C, germination timing was comparable between *R. alpinus* and *R. longifolius* (Figure 2e). Notably, 43% of *R. longifolius* seeds germinated synchronously on day 3 (Table S1), while 48% of *R. alpinus* seeds germinated on day 4. FG was highest for *R. alpinus* (88%) and *R. longifolius* (88%), with *R. obtusifolius* showing the lowest FG at 63% (Figure 2e).

At 35°C, *R. obtusifolius* exhibited poor germination performance, with FG reaching only 30% (Figure 2f). In contrast, *R. alpinus* and *R. longifolius* maintained relatively high FG values of 90% and 64%, respectively (Figure 2f). The majority of *R. alpinus* seeds germinated rapidly within the first 3 days, while *R. longifolius* followed on day 4. *R. obtusifolius* demonstrated a more gradual germination pattern (Table S1).

The fitted sigmoidal model highlighted distinct germination strategies across species. *R. alpinus* demonstrated stable germination rates across temperatures, with FG remaining consistently high (Figure 3a). In contrast, *R. obtusifolius* showed greater sensitivity to temperature variation, with germination success declining at 35°C (Figure 3b). The most pronounced differences were observed in *R. longifolius* , where FG varied substantially despite similar initial GR at different temperatures (Figure 3c).

To statistically confirm these differences in germination responses at varying temperatures, a Repeated Measures ANOVA was performed (Figure 3a–c). The analysis revealed significant differences in seed germination across temperature treatments for all studied species: *R. alpinus* ((*F*_145,696_) = 44.06, *p* < 0.001), *R. obtusifolius* ((*F*_145,667_) = 23.04, *p* < 0.001), and *R. longifolius* ((*F*_145,696_) = 20.95, *p* < 0.001).

### 3.2 Locality Germinated Seeds Experiment of *R. alpinus*

**FIGURE 4:**
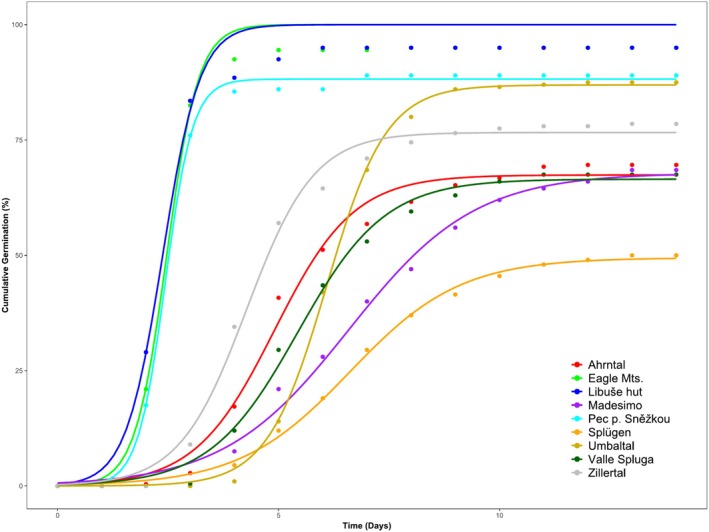
Cumulative germination (%) of *Rumex alpinus* from nine different localities over 14 days. Points indicate mean cumulative germination values, while solid lines represent fitted sigmoidal growth curves, modeled using a three‐parameter logistic function. Different colors represent different localities: Czech Republic (Eagle Mountains, Libuše hut, Pec pod Sněžkou), Austria‐Tyrol (Umbaltal, Zillertal), Italy‐Lombardy (Ahrntal, Madesimo, Val di Spluga), and Switzerland‐Lombardy (Splügen).

**FIGURE 5:**
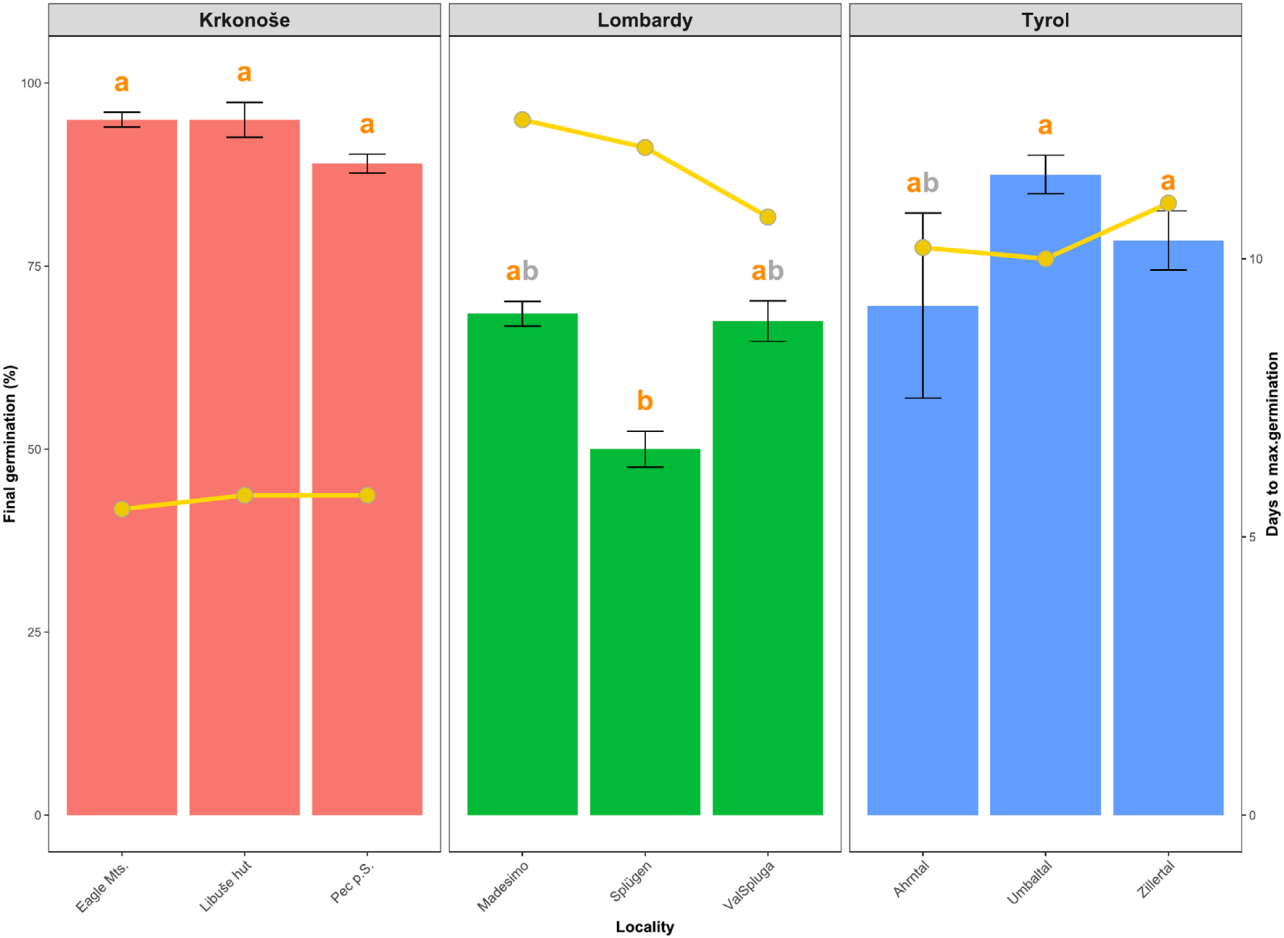
Final germination percentages and the number of days required to reach maximum germination for *R. alpinus* seeds collected from nine sampling localities across three mountain regions. Bars represent the mean final germination on day 14, with error bars indicating the standard error of the mean. Different letters above the bars denote statistically significant differences between localities based on Tukey's HSD test (*p* < 0.05). The gold line (right axis) shows the average number of days required to reach maximum germination for each locality. Mountain abbreviations: Eagle Mountains (Eagle Mts.), Val di Spluga (ValSpluga), and Pec pod Sněžkou (Pec p.S.).

The germination dynamics of *R. alpinus* differed significantly among localities, as illustrated in Figure 4. A repeated measures ANOVA revealed a significant interaction between time and locality (*F*_112,392_ = 16.570, *p* < 0.001). Additionally, a one‐way ANOVA (Figure 5) confirmed significant differences in final germination (FG) among all localities (*F*_8,28_ = 7.369, *p* < 0.001).

In the Czech mountain localities, GR and FG values were generally higher compared to the Alpine localities (Figures 4 and 5). The results showed that the highest GR was recorded on the second day (Table 3) in the Eagle Mountains, Pec pod Sněžkou, and Libuše hut, with all seeds reaching final germination by the fifth day (Figures 4 and 5). The average FG at Czech localities exceeded 93%, with the highest values recorded at Libuše hut (95%), followed by the Eagle Mountains (93%), and the lowest at Pec pod Sněžkou (89%) (Figure 5).

**TABLE 3:** The germination rate (GR) speed (mean% ± SE) of seeds from different localities.

Locality	1st day	2nd day	3rd day	4th day	5th day	6th day	7th day	8th day	9th day	10th day
Libuše hut	32.2 ± 2.64b*	**60.6 ± 4.20**b*	5.6 ± 0.64ab*	4.4 ± 1.57abc*	2.8 ± 1.40ab*	0.0 ± 0.00a*	0.0 ± 0.00a*	0.0 ± 0.00a*	0.0 ± 0.00a*	0.0 ± 0.00a
Pec pod Sněžkou	19.4 ± 5.84b	**65.0 ± 4.66**b	10.6 ± 2.10ab	0.56 ± 0.56a	0.0 ± 0.00a	3.3 ± 2.64ab	0.0 ± 0.00a	0.0 ± 0.00a	0.0 ± 0.00a	0.0 ± 0.00a
Eagle Mountains	23.3 ± 5.84b	**68.3 ± 6.11**b	11.1 ± 0.91ab	2.2 ± 0.91ab	0.0 ± 0.00a	0.0 ± 0.00a	0.6 ± 0.56ab	0.0 ± 0.00a	0.0 ± 0.00a	0.0 ± 0.00a
Ahrntal	0.5 ± 0.44a	2.7 ± 1.30a	16.0 ± 5.28bc	**26.2 ± 10.69**c	11.6 ± 4.00ab	6.2 ± 2.93ab	5.3 ± 1.66abc	4.0 ± 1.47ab	1.8 ± 0.44a	2.7 ± 1.78a
Umbaltal	0.0 ± 0.00a	0.0 ± 0.00a	1.1 ± 0.64a	14.4 ± 2.13abc	**31.1 ± 5.44**c	29.4 ± 3.06c	12.8 ± 3.19d	6.7 ± 2.87ab	0.6 ± 0.56a	0.6 ± 0.56a
Zillertal	0.0 ± 0.00a	10.0 ± 1.11a	**28.3 ± 3.44**c	25.0 ± 3.32bc	8.3 ± 2.78ab	7.2 ± 1.90ab	3.9 ± 0.56abc	2.2 ± 0.91a	1.1 ± 0.64a	0.6 ± 0.56a
Madesimo	0.0 ± 0.00a	0.0 ± 0.00a	8.3 ± 1.40ab	**15.0 ± 1.40**abc	7.8 ± 1.43ab	13.3 ± 1.57b	7.8 ± 1.11bcd	10.0 ± 2.80b	6.7 ± 2.03b	2.8 ± 0.56a
Splügen	0.0 ± 0.00a	0.0 ± 0.00a	5.0 ± 1.67ab	8.3 ± 2.92abc	7.8 ± 2.31ab	**11.7 ± 2.92**b	8.3 ± 1.40 cd	5.0 ± 0.56ab	4.4 ± 1.81ab	2.8 ± 1.40a
Val di Spluga	0.0 ± 0.00a	0.6 ± 0.56a	12.8 ± 3.19ab	**19.4 ± 2.92**abc	15.56 ± 3.27b	10.6 ± 2.29ab	7.2 ± 1.90ad	3.9 ± 1.67ab	3.3 ± 1.43ab	1.7 ± 0.56a

*Note:* The *p‐*value was obtained by one‐way ANOVA. Using the Tukey post hoc test, the mean values of each day with the same letter among localities were not significantly different. Bold font highlights the percentage values with the highest number of germinated seeds.

* *p* < 0.01.

In contrast, GR was generally slower in the Alpine localities. For instance, in the Tyrolean Alps (Zillertal), the highest GR was observed on the third day, but final germination was reached only on the tenth day (Table 3, Figure 5). The average FG in Alpine localities was 70%, with the highest value recorded at Umbaltal (88%), followed by Zillertal (78%) and Ahrntal (73%).

Even lower FG values were observed in the Lombard Alpine localities, where the average FG was 70%, with the lowest recorded value of 50% in Splügen. Final seed germination in the Alpine localities generally occurred around the tenth day (Figure 5).

## 4 Discussion

Detailed studies of generative reproduction have proven to be valuable tools, providing important insights into the determinants of species invasiveness (Vojík et al. 2022). Research into the germination traits of plant taxa (Grime et al. 1988), for instance, aids in understanding their capacity for spreading and the likelihood of establishing new populations (Pyšek and Richardson 2007).

In the laboratory experiment, the germination rates (GR) and final germination (FG) of three *Rumex* spp. were investigated at different temperatures. The results indicated that temperature significantly influences *Rumex* spp. germination, but cannot fully explain their distribution at different altitudes. Despite the well‐documented deep summer dormancy of *R. obtusifolius* (Cavers and Harper 1964; Benvenuti et al. 2001; Müllerová 2015; Suter et al. 2023), the observed responses following anti‐dormancy treatment suggested similar mechanisms across all species. Nevertheless, *R. obtusifolius* and *R. alpinus* were germinated at 6°C, whereas *R. longifolius* did not. It can be concluded that low temperatures were not the critical factors controlling seed germination for either species (Totterdell and Roberts 1979). This contrasts with the findings of Bhatt et al. (2023), who reported that *R. obtusifolius* did not germinate under low temperatures (5°C/10°C).

Germination timing depends on a combination of factors, including light and temperature (Hoyle et al. 2015), as well as macronutrient availability in the soil (Hrdličková et al. 2011). External temperature and light availability directly influence enzymes involved in the germination process (Baskin and Baskin 1998). In this study, *R. longifolius* exhibited a delayed GR and a lower FG at temperatures of 6°C and 12°C. This species likely requires a specific temperature range to activate the enzymes necessary for germination, as cold temperatures can impede or slow enzyme activation, leading to reduced germination rates (Bihzad and El‐Shora 1996). Furthermore, low temperatures can prolong seed dormancy (Van Assche et al. 2002), reduce metabolic rates (Chidananda et al. 2014), and inhibit the hydrolysis of reserves required for germination.

Notably, *R. alpinus* exhibited the highest FG and nearly the highest GR across all tested temperature regimes, suggesting that it may be more plastic and tolerant to environmental conditions than the other studied species. This aligns with the findings of Šťastná et al. (2010) and Doležal et al. (2020). The slower FG and GR observed for *R. obtusifolius* at the lowest temperatures are consistent with previous findings by Cavers and Harper (1964). However, a change occurred at 18°C when seeds of all three species started germination at approximately the same time. Nonetheless, *R. alpinus* and *R. longifolius* exhibited higher FG and GR than *R. obtusifolius* . Our findings suggest that species originating from regions with a limited growing season may exhibit differences in germination timing, which warrants further investigation (Holm and Korpelainen 1999; Kubínová and Krahulec 1999). This could be further supported by our findings, which revealed a high germination percentage for *R. longifolius* only at elevated temperatures.

The optimal temperature for germination of *Rumex* spp. was approximately 24°C, consistent with the findings of Benvenuti et al. (2001) and Le et al. (2021). Notably, *R. longifolius* exhibited the highest GR, followed by *R. alpinus* , whereas *R. obtusifolius* germinated more slowly over several days. The FG of *R. alpinus* and *R. longifolius* reached approximately the same level at 29°C, which contrasts with the findings of Van Assche et al. (2002), who found that *R. longifolius* did not germinate as extensively at this temperature. However, our results align with those of Moravcová et al. (2010), who reported FG values of 86% and 92% for *R. alpinus* and *R. longifolius* , respectively.

It is noteworthy that the FG of *R. obtusifolius* did not differ significantly within the temperature range of 12°C to 24°C, suggesting a broader temperature optimum than previously reported by Benvenuti et al. (2001) and Le et al. (2021). This finding also supports the results of Park et al. (2010), who identified an optimum temperature of 15°C, with higher temperatures significantly reducing germination. It is further possible that seeds from different parts of the same inflorescence may exhibit variation in dormancy, with seeds from the proximal branches of the inflorescence tending to have higher dormancy levels than those from distal positions (Martinková and Honěk 2001; Honěk and Martinková 2002).

At 35°C, *R. longifolius* and *R. obtusifolius* exhibited suboptimal germination. Although *R. longifolius* initiated germination at approximately the same time as *R. obtusifolius* , it reached a higher FG. Overall, *R. obtusifolius* demonstrated robust germination capacity, except at the highest temperature (35°C). However, its ability to germinate effectively over a wide range of temperatures was expected, given the species' capacity to thrive in diverse climatic conditions, including those found in tropical, subtropical, and temperate ecosystems (Bhatt et al. 2023). Nevertheless, the germination rate could have been higher if the laboratory conditions had more closely simulated the complex interactions occurring in the natural environment. In their native habitat, seeds are exposed to numerous environmental factors, including fluctuations in temperature, humidity, and light, as well as interactions with other organisms (Klupczyńska and Pawłowski 2021; Bhatt et al. 2023).

Seeds of *R. longifolius* were collected from its native habitat in Iceland due to the lack of available seeds in the Czech Republic. Similarly, seeds of *R. obtusifolius* were collected in the Czech Republic, where the species is native, while *R. alpinus* seeds were collected in the Czech Republic, where it is not native. Consequently, a comparative analysis was necessary to assess the significance of this factor. Such comparisons are crucial for elucidating patterns of range expansion and adaptation processes (Donohue et al. 2005). Therefore, a second experiment was conducted to investigate differences in germination of *R. alpinus* seeds from native (Alps) and non‐native (Czech Republic) localities (Jungová, Jurasová Müllerová, et al. 2023). The experiment aimed to examine the impact of non‐native and native habitats on seed germination. The results indicated significant differences in the germination rates (FG and GR). Notable variations were observed in the germination rate according to geographical location, with a positive correlation between increasing latitude and germination performance. Seeds originating from locations within the Krkonoše Mountains, where *R. alpinus* is an introduced and invasive species, exhibited the fastest GR and highest FG. This difference may be attributed to the species' need to rapidly colonize non‐native habitats, resulting in accelerated germination (Kutschera and Lichtenegger 1992; Bucharová 2003; Červenková and Münzbergová 2009).

The rapid germination strategy (Gioria and Pyšek 2017) is particularly prevalent in ornamental plants, where it may be explained by adaptation to artificial habitats in urban areas, which strongly influences the adaptation of species (Williams et al. 2015; McDonnell and Hahs 2015). Furthermore, species often modify their ecological and reproductive traits to enhance invasiveness, with rapid germination strategies likely to become more prevalent (Dubois and Cheptou 2017). A similar strategy has been observed in *Heracleum mantegazzianum* (Gioria and Osborne 2009) and the invasive lobed ragweed, *Ambrosia artemisiifolia* (Gorton et al. 2018). Similarly, our present results are also consistent with those of Moravcová et al. (2010).

Although *R. alpinus* seeds from non‐native localities exhibited the highest germination, this pattern could be attributed to multiple factors, including potential adaptability to new environmental conditions (Williams et al. 2015; Zhang et al. 2020), maternal effects (Klupczyńska and Pawłowski 2021), or differences in dormancy status. However, in this study, all seeds underwent cold stratification at 5°C for 6 weeks before germination experiments, a method commonly used to break potential dormancy (Mandák et al. 2006; Kołodziejek 2019). Although previous research (Pavlů, pers. comm.) and our own experience (Martinková and Honěk 2001; Honěk and Martinková 2002) indicate that *Rumex* spp. do not exhibit strong dormancy and germinate readily under suitable conditions (Šťastná et al. 2010; Hujerová 2010; Hujerová et al. 2013, 2017), the applied stratification treatment was designed to standardize initial conditions and mitigate any residual dormancy effects. Given this, it is unlikely that dormancy played a major role in the observed germination differences.

Additionally, alongside these experiments, we conducted a study on seedlings grown from these germinated seeds in a climate chamber (data not yet published). The results indicate that *R. alpinus* seedlings exhibit significantly slower growth than *R. longifolius* and *R. obtusifolius* , suggesting that further research is needed to determine whether this trait contributes to its absence at lower elevations. *Rumex alpinus* is considered a C‐strategist, characterized by slower and more consistent growth rates (Grime 1979; Klotz and Kühn 2002; Chytrý et al. 2021), which may limit its ability to establish in more competitive lowland environments. Its seedlings are competitively weaker in habitats dominated by certain grass species (Jiřiště 2000; Červenková and Münzbergová 2009; Náglová 2014), potentially explaining the observed rapid germination as a compensatory mechanism (Kahl et al. 2019).

However, this study provides an exploratory insight into the germination behavior of *R. alpinus* across populations from different localities, highlighting the need for further controlled experiments, including common garden studies and additional seed dormancy assessments, to fully isolate the role of temperature from other potential influences.

Another factor that may contribute to germination variability is the potential negative effect of maternal plants, which could inhibit seed germination. Some studies suggest that the presence of certain invasive species, such as *Reynoutria japonica* , suppresses the germination of other plant species through allelopathic interactions (Mikulic‐Petkovsek et al. 2022; Kadlecová et al. 2024). Thus, it is conceivable that *R. alpinus* may also inhibit the germination of its own seeds, although this hypothesis remains to be tested. Additionally, genetic growth constraints or adverse environmental conditions, such as cold winter temperatures, may act as selective pressures on early seedling cohorts, determining which individuals survive regardless of their ploidy levels (Saad et al. 2011). Further research is necessary to assess whether these factors contribute to the observed differences in germination success and seedling establishment in *R. alpinus* .

This suggests that factors beyond temperature may also be critical in determining the establishment success of *R. alpinus* . Furthermore, its ability to reproduce vegetatively under unfavorable conditions (Doležal et al. 2020) may play a significant role in its persistence, irrespective of seed‐based establishment (Klimeš 1992; Klimeš et al. 1993; Šťastná et al. 2012; Náglová 2014; Šilc and Gregori 2016).

## 5 Conclusion

Temperature significantly influenced the germination of all tested *Rumex* species. *Rumex alpinus* exhibited the highest germination rate, suggesting that this species possesses greater plasticity and tolerance to local environmental conditions than the other species studied.

The results demonstrated that *R. longifolius* , which is adapted to colder conditions, exhibited lower germination rates at lower temperatures, whereas *R. obtusifolius* , adapted to warmer conditions, displayed reduced germination rates under these conditions. However, it is important to acknowledge that high temperatures are not the sole factor limiting the inability of *R. alpinus* to establish in lowland areas. Further research is therefore required to investigate additional environmental constraints. To prevent the further spread of this species, appropriate management measures should be considered.

A significant geographic variation in the germination rates of *R. alpinus* was observed, with higher rates in non‐native localities and lower rates in native Alpine localities. This may be attributed to the species' plasticity in response to new environmental conditions. Our findings provide insights into temperature‐driven germination differences in *Rumex* spp., forming a basis for further research into their environmental limitations. As suggested by other studies, the degree of seed shading in the environment may also play a significant role in germination success.

Consequently, further investigations are necessary to identify additional factors influencing species distribution and establishment.

## Ethics Statement

The authors have nothing to report.

## Conflicts of Interest

The authors declare no conflicts of interest.

## Supporting information


Table S1.

## Data Availability

The data supporting this study's findings are available from the corresponding author upon request.
