# Supplementary material for: Ecological Implications of Germination Temperature on Native and Invasive Rumex Spp
Source: Plant Environ Interact. 2025 Mar 27;6(2):e70045. doi: 10.1002/pei3.70045 (PMC11949848; doi:10.1002/pei3.70045)
Supplement: Supplementary file 1 — Table S1. [file PEI3-6-e70045-s001.docx]

**Ecological implications of germination temperature on native and invasive *Rumex* spp.**

Michaela Jungová^1^, Martina Kadlecová^2^, Vilém Pavlů^1,3^, Leona Leišová-Svobodová^1^, Pavel Svoboda and^1^and Zdenka Martinková^1^

^1^Czech Agrifood Research Center, Drnovská 507/73, 161 06 Prague 6 – Ruzyně, Czech Republic

^2^Department of Applied Ecology, Faculty of Environmental Sciences, Czech University of Life Sciences Prague, Kamýcká 129, CZ 165 21 Prague 6 – Suchdol, Czech Republic

^3^Department of Biology and Ecology, Technical University of Liberec, Rolnická 6, 460 11 Liberec, Czech Republic

Correspondence

Michaela Jungová, Drnovská 507/73, 161 06 Prague 6 – Ruzyně, Czech Republic

Email: michaela.jungova@carc.cz; <https://orcid.org/0000-0003-1445-5981>

**Table S1** The germination rate (GR) (mean% ± SE) of seeds for *R. alpinus, R. obtusifolius,* and *R. longifolius*. The *p-*value was obtained by one-way ANOVA. Asterisk (*) denotes the level of statistical significance *p* < 0.01. Using the Tukey *post hoc* test, the mean values of each day with the same letter among *Rumex* species were not significantly different. Bold font highlights the percentage values with the highest number of germinated seeds for each *Rumex* spp. The table of GR at different temperatures is provided only for the days when germination was faster, specifically days 1 to 10 for temperatures ranging from 12 to 35°C. As germination occurred later at 6°C, it was not included in the table.

| **Days** | **1** | **2** | **3** | **4** | **5** | **6** | **7** | **8** | **9** | **10** |  |
| --- | --- | --- | --- | --- | --- | --- | --- | --- | --- | --- | --- |
| *R. alpinus* | 0.0±0.00**a** | 0.0±0.00**a*** | 2.1±0.66**a** | 5.8±2.32**a** | 14.6±4.52**b*** | **32.5±3.76b*** | 26.3±5.34**b*** | 2.5±1.67**ab*** | 4.2±1.14**b*** | 1.3±0.83**a*** | **12°C** |
| *R. obtusifolius* | 0.0±0.00**a** | 0.0±0.00**a** | 0.8±0.51**a** | 0.0±0.00**a** | 0.4±0.42**a** | 3.8±1.38**a** | **20.0±2.68b** | 5.0±0.78**b** | 6.7±0.78**b** | **12.1±3.51b** |  |
| *R. longifolius* | 0.0±0.00**a** | 2.1±0.93**b** | **4.6±1.67a** | 3.3±1.69**a** | 4.2±1.47**a** | 0.42±0.42**a** | 0.4±0.42**a** | 0.42±0.42**a** | 0.00±0.00**a** | 0.42±0.42**a** |  |
| *R. alpinus* | 2.0±1.19**a** | 24±1.95**b*** | **60.0±5.38b*** | 18.5±2.51**a*** | 3.9±1.46**ab*** | 0.5±0.49**a** | 0.0±0.00**a** | 0.5±0.49**a** | 0.5±0.49**a** | 0.0±0.00**a** | **18°C** |
| *R. obtusifolius* | 0.5±0.49**a** | 1.0±0.60**a** | 24.0±3.40**a** | **46.8±6.14b** | 9.7±2.99**b** | 0.5±0.49**a** | 0.0±0.00**a** | 1.5±0.60**a** | 1.0±0.60**a** | 0.5±0.49**a** |  |
| *R. longifolius* | 1.0±0.60**a** | **25.4±3.58b** | 12.7±2.36**a** | 7.3±2.67**a** | 0.5±0.49**a** | 0.0±0.00**a** | 0.5±0.49**a** | 1.0±0.60**a** | 0.5±0.49**a** | 0.5±0.49**a** |  |
| *R. alpinus* | 0.0±0.00**a*** | 12.2±2.96**a** | 16.10±1.90**a*** | **50.4±3.25b*** | 10.4±3.53**a** | 2.6±0.81**a** | 1.3±0.53**a*** | 0.4±0.43**a*** | 0.0±0.00**a*** | 0.0±0.00**a** | **24°C** |
| *R. obtusifolius* | 0.4±0.43**a** | 8.7±2.06**a** | **11.7±2.24a** | 10.4±1.06**a** | 9.6±2.54**a** | 8.7±2.28a | 5.2±0.53b | 7.0±1.27**b** | 3.5±0.90**b** | 1.7±0.81**a** |  |
| *R. longifolius* | 4.4±1.19**b** | 13.1±3.70**a** | **27.8±2.32b** | 16:1±1.30**a** | 7.0±2.11**a** | 3.0±2.02**a** | 0.0±0.00**a** | 3.9±1.06**ab** | 3.9±0.43**b** | 0.9±0.53**a** |  |
| *R. alpinus* | 7.7±1.36**b*** | 8.2±1.85**a** | 16.4±1.51**b*** | **48.2±1.33b*** | 12.3±3.18**a** | 5.5±1.16**b*** | 0.9±0.56**a*** | 0.0±0.00**a** | 0.9±0.56**a** | 0.0±0.00**a** | **29°C** |
| *R. obtusifolius* | 1.4±0.56**a** | 9.6±1.96**a** | 3.6±1.54**a** | **15.5±1.67a** | 14.1±2.20**a** | 6.8±1.24**b** | 8.6±0.86**b** | 2.3±0.72**a** | 2.3±0.72**a** | 0.5±0.45**a** |  |
| *R. longifolius* | 3.2±1.36**a** | 4.6±1.24**a** | **43.2±4.77c** | 16.4±3.32**a** | 10.5±2.11**a** | 1.4±0.56**a** | 1.8±0.85**a** | 1.8±0.85**a** | 1.4±0.91**a** | 1.4±0.56**a** |  |
| *R. alpinus* | 22.4±2.72**b*** | **42.0±4.52b*** | 37.6±5.85**b*** | 6.3±2.74**ab*** | 0.5±0.49**a*** | 0.5±0.49**a** | 0.5±0.49**a** | 0.0±0.00 | 0.0±0.00 | 0.0±0.00 | **35°C** |
| *R. obtusifolius* | 2.4±0.77**a** | 3.4±1.24**a** | 7.3±2.04**a** | 4.4±0.91**a** | **9.3±2.93ab** | 6.3±2.13**a** | 2.9±0.91**a** | 0.0±0.00 | 0.0±0.00 | 0.0±0.00 |  |
| *R. longifolius* | 4.9±1.34**a** | 6.8±1.95**a** | 16.1±6.57**a** | **23.0±7.42b** | 12.2±4.08**b** | 5.4±1.95**a** | 7.3±4.29**a** | 0.0±0.00 | 0.0±0.00 | 0.0±0.00 |  |
